# Novel insights into the emerging roles of tRNA-derived fragments in mammalian development

**DOI:** 10.1080/15476286.2020.1732694

**Published:** 2020-03-01

**Authors:** Nicola Guzzi, Cristian Bellodi

**Affiliations:** Division of Molecular Hematology, Department of Laboratory Medicine, Lund Stem Cell Center, Faculty of Medicine, Lund University, Lund, Sweden

**Keywords:** tRNA fragments, RNA modifications, RNA epitranscriptomics, translation control, stem cells, development, hematopoiesis, pseudouridine, 5-methylcytosine

## Abstract

tRNA-derived fragments or tRFs were long considered merely degradation intermediates of full-length tRNAs; however, emerging research is highlighting unanticipated new and highly distinct functions in epigenetic control, metabolism, immune activity and stem cell fate commitment. Importantly, recent studies suggest that RNA epitranscriptomic modifications may provide an additional regulatory layer that dynamically directs tRF activity in stem and cancer cells. In this review, we explore current work illustrating unanticipated roles of tRFs in mammalian stem cells with a focus on the impact of post-transcriptional RNA modifications for the biogenesis and function of this growing class of small noncoding RNAs.

## Introduction

Recent advances in high-throughput RNA sequencing methods and comprehensive analysis of the small RNA compartment [1–4] have revealed myriad evolutionarily conserved non-coding (nc) RNA populations that are derived from various RNA species such as ribosomal RNA [5], messenger RNA [6], vault RNA [7], Y RNA [8] and transfer RNA (tRNA), also referred to as tRNA fragments (tRFs or tsRNAs) [9–14]. Although tRNA cleavage events were recognized in the body fluid of cancer patients already as early as the late 1970s [15,16], the functional potential of these tRNA processing intermediates has remained overlooked until recently. Intriguingly, emerging findings suggest that specific tRF subsets may harbour unanticipated biological activity and dynamically impact genetic information in mammalian cells. Taken together these results have sparked new interest into this expanding area of tRNA research.

tRF classification is based on their original location on the corresponding tRNA isoacceptor in three major categories: 5ʹ-derived tRFs (5ʹ-tRF), 3ʹ-derived tRFs (3ʹ-tRF) and internally derived tRFs (i-tRF) [17,18]. In addition, tRFs can be further divided in constitutive (steady-state) and stress-induced fragments, which are characterized by heterogeneous sequence and length distribution [13]. It has been shown that short 15–22 nucleotide (nt) long fragments are constitutively produced across several tissues and cell lines even in homeostatic conditions [19], whereas longer 31–40 nt tRNA-halves are predominantly generated in response to various stressors [3,4,20]. Notably, only a minor portion of mature cytoplasmic tRNAs (~1%) is processed during stress [4], strongly suggesting that stress-induced tRNA cleavage is unlikely to deplete cellular tRNA pools.

**Figure 1.**
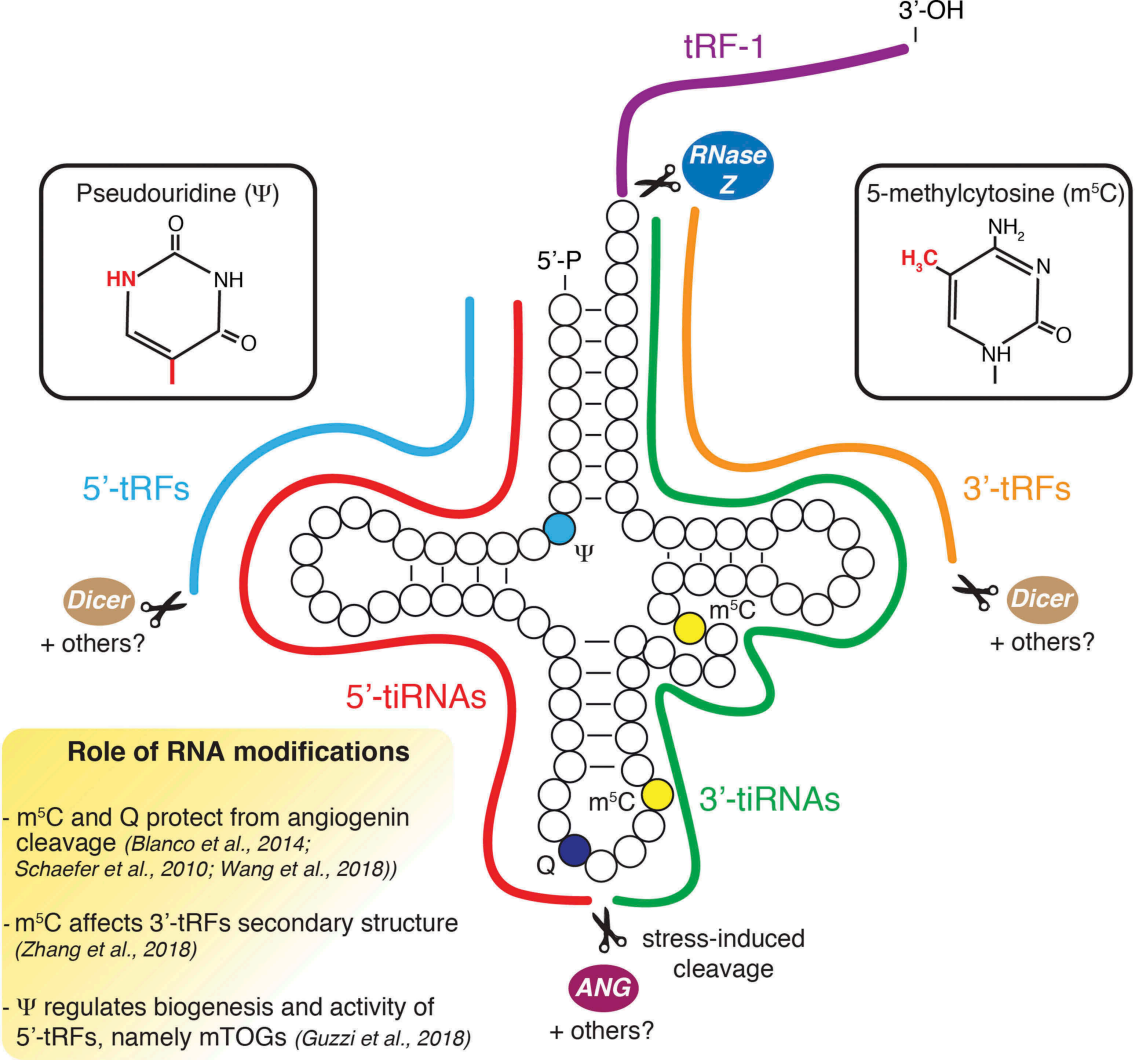
**Regulation of tRF biogenesis in stem cells.** Schematic illustrates tRF biogenesis and epitranscriptomic modifications. Dicer and possibly other ribonucleases not yet identified have been proposed to modulate the production of 5ʹ- and 3ʹ-tRFs. Angiogenin (ANG) induces cleavage of tRNAs in the anticodon loop in response to stress conditions to promote the accumulation of 5ʹ- and 3ʹ-tiRNAs. 5-methylcytosine (m^5^C) and queuosine (Q) modulate angiogenin activity and may impact 3ʹ-tRF secondary structure. Pseudouridine (ψ) is necessary for the biogenesis and activity of a specific class of 5ʹ-tRFs (mTOGs) in stem cells.

A breakthrough came with the identification of the vertebrate-specific RNase superfamily member angiogenin, which is responsible for the production of stress-induced tRNA-derived fragments (tiRNAs) in mammalian cells [1,4]. Furthermore, studies using RNA methyltransferases knockout cells and mouse models revealed that the ability of angiogenin to cleave tRNAs was modulated by the presence of 5-methylcytosine (m^5^C) base modification [21,22], unravelling a critical role of RNA modifications in tRF biology. This additional regulatory layer of tRF biogenesis was further supported by findings that queuosine modification at position 34 modulated angiogenin-dependent cleavage [23]. Over 90 different types of base modifications decorate tRNAs in humans with an average of 11–13 modifications per molecule, which provides plenty of room for post-transcriptional regulation of tRFs [24]. The importance of RNA modifications for tRF biogenesis and function has increasingly been appreciated and was recently reviewed [25]. Additionally, an important functional overlap with the miRNA pathway was proposed with findings that Dicer might be involved in the accumulation of 5ʹ- and 3ʹ-tRFs [9,26]. However, the role of Dicer in tRF biogenesis may be limited, as its depletion does not globally impact tRF levels in multiple model organisms [19,27,28]. Furthermore, other endonucleases have been shown to target tRNAs in mammalian cells. For example, the RNase Z/ELAC2 was involved in pre-tRNAs fragmentation to generate a specific class of 3ʹ-derived tRFs (tRF-1) with a unique sequence not present in mature tRNAs [13]. Another study illustrated that RNase L activation in response to viral infection led to site-specific tRNA cleavage and translational repression in human cancer cells, although whether the resulting tRFs harboured biological activity was not explored [29]. Given the complexity of the processing pathways involved in tRNA fragmentation, dissecting how tRF biogenesis undergoes cell context-specific regulation represents one of the major current challenges in tRF biology (Fig. 1 and Outstanding Questions).

Recent research on the role of tRFs has highlighted important functions in a variety of molecular processes including mRNA stabilization [30], regulation of cap-dependent and cap-independent translation [4,20,31–37], miRNA-mediated silencing [2,26,38], mRNA localization in stress granules [39] and suppression of transposable elements (TEs) [40]. Moreover, tRF production is also dynamically regulated during fundamental cellular processes involved in development and commonly dysregulated in cancer, thus increasing the functional complexity of these small RNAs [34,41]. These findings implicate tRFs as integral molecular components of gene expression programs that may contribute to direct lineage-specific commitment. This raises an important question: what is the underlying regulatory potential of tRFs in stem cells? In this review, we will summarize recent research highlighting the physiological importance of tRFs in germline, embryonic and somatic stem cells with a focus on the impact of RNA epitranscriptomic modifications on the biogenesis and function of specific tRF subsets during mammalian development.

## tRFs in epigenetic regulation and germline-mediated intergenerational inheritance

**Figure 2.**
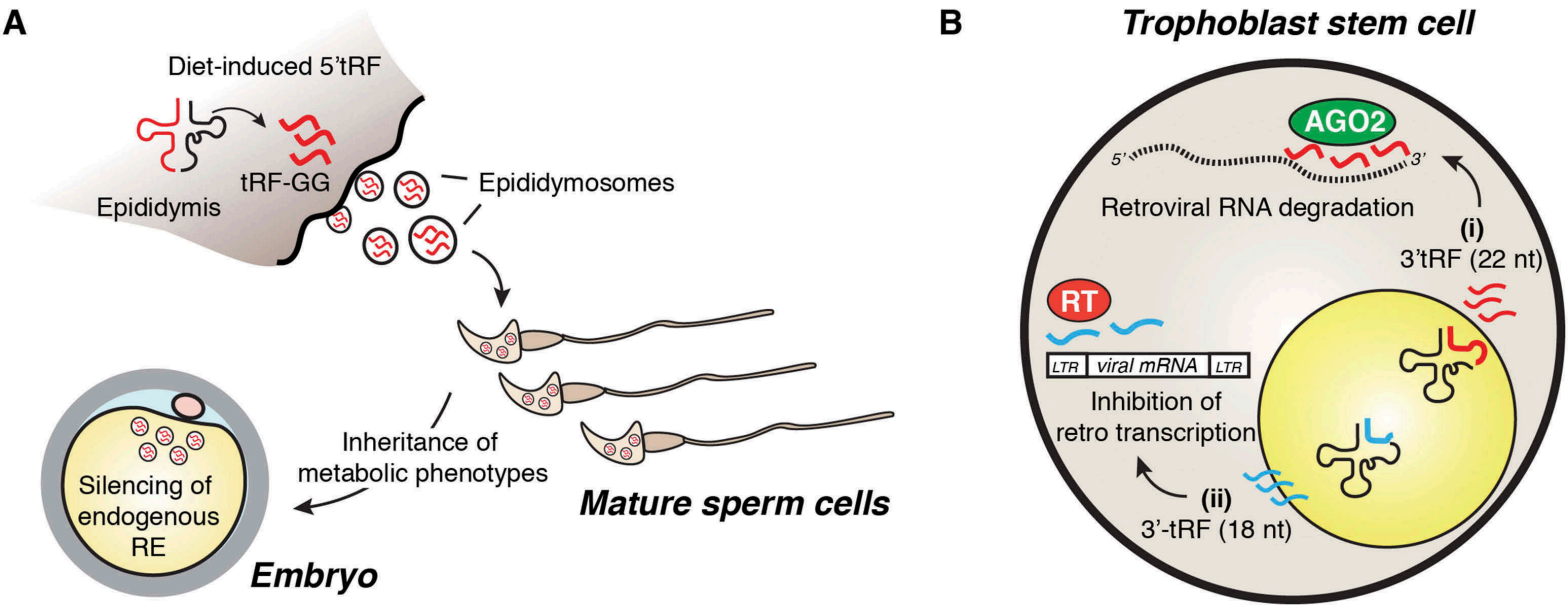
**tRF function in epigenetic silencing during early embryogenesis.** (A) tRFs mediate intergenerational transmission of metabolic phenotypes. 5ʹ-tRFs including tRF-GG, exhibit a two-three fold increase in somatic epididymis cells upon dietary restriction and are transferred to maturing sperm cells. This process has been proposed to repress endogenous retroelements (RE) and pass paternal inherited information during embryonic development. (B) Murine trophoblastic stem cells produce 3ʹ-tRFs that inhibit the replication of endogenous retroviruses via two distinct mechanisms: (i) putative model illustrating 22 nt 3ʹ-tRFs inducing post-transcriptional silencing of retroviral RNA possibly via association with the canonical miRNA-effector protein AGO2. (ii) 18 nt 3ʹ-tRFs interfere with reverse transcription of viral RNAs to restrict transposon mobility.

Several lines of evidence indicate a function for tRFs in gene expression control during early embryonic development. Seminal work demonstrated that epigenetic DNA modifications alone could not explain male contribution to intergenerational inheritance traits and indicated that RNA may be central to this process [42]. Additional studies further involved sperm tRFs in promoting intergenerational transmission of specific metabolic phenotypes in mice [43,44]. Authors showed that mature sperm cells were characterized by an abundant 5ʹ- and 3ʹ-tRF payload that was altered in response to environmental cues such as proteinrestriction and high-fat diet (HFD) regimens. Indeed, sperm 5ʹ-tRF levels were significantly different in mice fed with a low-protein diet compared to normal-diet controls and may contribute to transmission of metabolic phenotypes through inhibition of TE [44]. Unexpectedly, low levels of tRFs were detected in immature sperm cells purified from mice testicles. On the basis of this observation, it was subsequently shown that sperm cells were able to acquire their tRF payload after testicular maturation through fusion of transport vesicles enriched in 5ʹ-tRFs produced in the epididymis (epididymosomes) [45]. This compellingly suggested that tRF pools might be transferred between different cell types to pass higher-order organismal information, such as dietary protein restrictions, through the germline (Fig. 2A).

Additional evidence illustrated that a significant proportion (~11.5%) of mature sperm 5ʹ- and 3ʹ-tRFs were differentially produced in mice kept on a HFD compared to a normal diet [43]. Remarkably, tRF-enriched small RNA fractions derived from HFD sperm were sufficient to induce glucose intolerance in the offspring when transferred to normal zygotes [43]. Despite these findings, the exact mechanisms that account for tRF-mediated intergenerational inheritance remain to be fully elucidated. Interestingly, zygotic injection of synthetic tRFs failed to recapitulate HFD-driven metabolic disorder phenotypes in the offspring [43], further indicating that the stability and activity of sperm-derived endogenous tRF pools might rely on post-transcriptional modifications [46]. This idea was further supported by findings that the levels of m^5^C and N2-methylguanosine (m^2^G) base modifications were significantly elevated within sperm small RNAs (30–40 nt) derived from HFD fed mice [43,46]. These studies suggest that distinct RNA modifications may impact the biogenesis and function of sperm-derived tRFs, thereby contributing to the transmission of metabolic phenotypes in the offspring. Consistently, subsequent results indicated that HFD induced the expression of the RNA methyltransferase Dnmt2/Trdmt1 hereafter referred to as Dnmt2, in the epididymis [46]. Critically, it was previously reported that Dnmt2-dependent m^5^C at position 38 protected specific tRNAs from angiogenin-mediated cleavage [22,47]. Thus, it is possible that HFD-dependent induction of Dnmt2 levels may be required to fine-tune sperm tRF cargos and promote inheritance of paternal phenotypic traits. Accordingly, genetic depletion of *Dnmt2* in mouse models altered tRF profiles in sperm cells and prevented the transmission of paternally acquired metabolic disorders [46]. This study further revealed that Dnmt2-mediated m^5^C affected the secondary structure and biological properties of abundant 3ʹ-tRFs upon transfection in cell lines [46], although the exact contribution of m^5^C for tRF-mediated paternal inheritance was not fully delineated. Similarly, a recent study in humans showed that i-tRF levels in sperm cells from healthy donors were dynamically modulated in response to acute diet intervention and were positively associated with increased sperm motility [48]. Hence, further studies will be required to qualitatively assess the function of tRFs during the early developmental stages and their contribution to the transmission of paternally acquired metabolic traits.

In addition to a putative role in paternal inheritance, tRFs have been shown to strongly inhibit endogenous TEs [40]. Interestingly, extra-embryonic trophoblast and embryonic stem cells (ESC) with de-repressed chromatin were characterized by remarkably high levels of 3ʹ-tRFs, thus suggesting a putative role for these small ncRNAs in controlling transposon mobility [40]. Two possible mechanisms have been proposed for tRF-mediated retrotransposon silencing: (i) short 18 nt 3ʹ-tRFs interfere with reverse transcription by targeting the reverse transcriptase primer binding sites; (ii) 22 nt 3ʹ-tRFs induce post-transcriptional silencing of viral RNA [40] possibly via association with Argonaute 2 (AGO2) as previously proposed [28] (Fig. 2B). However, direct evidence for tRF-driven AGO2 silencing is still lacking. Novel mechanistic insights into how tRFs may control TE were recently revealed in a new study. Authors found that a specific 5ʹ-tRF derived from tRNA-Gly-GCC, tRF-GG, regulated the expression of the endogenous retroelement MERVL in mouse ESCs (mESC) and in pre-implantation embryos [49]. Strikingly, tRF-GG promoted the biogenesis of a wide range of ncRNAs including U7 snRNA, which contributed to the stabilization of histone mRNAs through binding to a histone stem loop (HSL) located in the 3ʹ-untranslated region (UTR) of these transcripts. This subsequently resulted in heterochromatin assembly associated with TE silencing in mESCs. Since tRF-GG was previously highlighted as one of the most differentially produced fragments (two-three fold increase) in sperm cells upon protein restriction, it is possible that a similar mechanism may also be involved in the parental transmission of metabolic phenotypes [44]. Together, these findings indicate that tRFs may contribute to genomic stability during the earliest steps of development.

## Developmentally regulated tRF subsets impact embryonic stem cell function

Novel insights into the role of tRFs in embryogenesis were recently obtained using human ESCs (hESCs) [34]. This work unveiled a new molecular circuitry orchestrated by a pool of short 5ʹ-tRFs, denoted mini TOGs (mTOGs), that were characterized by a distinct 5ʹ terminal oligoguanine (TOG) motif shared by a group of tRNA isoacceptors including tRNA-Ala, tRNA-Cys and tRNA-Val [34]. We discovered an essential role for the stem cell-enriched pseudouridine synthase, PUS7, in the biogenesis and function of mTOGs in hESCs. Importantly, PUS7 mediated the pseudouridylation (ψ) of a critical uridine (U) at position 8 within the mTOG sequence and this was required for translation repression through selective binding and inhibition of the polyadenylate binding protein 1 (PABPC1), an integral component of the 5ʹ cap-translation initiation complex. Notably, PUS7-depleted hESCs were characterized by dramatic growth and differentiation defects that significantly impaired germ layer specification in the absence of changes in tRNA abundance. Findings that PUS7 and mTOGs were rapidly downregulated during embryonic differentiation further suggest that the post-transcriptional regulatory layer governed by tRFs and ψ may provide a physiological means to spatiotemporally control gene expression during development. As such, more work will be necessary to determine the underlying developmental signals and molecular mechanisms that direct PUS7-mediated ψ and mTOG repressive function in embryonic development. Further evidence that tRFs may contribute to lineage commitment of mouse ESCs (mESCs) was reported in a recent study [50]. Specifically, it was shown that the abundance of defined 5ʹ-tRFs was differentially modulated in mESCs undergoing retinoic acid (RA)-induced differentiation and this was uncoupled from parental tRNA isoacceptors gene copy number. Additionally, authors showed that only a small percentage of differentiation-induced tRFs were dependent on angiogenin processing [50]. This evidence suggested that additional RNases, other than angiogenin, might become activated in response to differentiation-promoting signals to modulate tRF abundance. Importantly, developmentally controlled 5ʹ-tRFs were shown to repress the expression of key pluripotency factors including *c-Myc* and *Klf4*. Specifically, authors found that the effect on c-*Myc* levels was in part mediated through the direct association between a 35 nt 5ʹ-tRF (tsGlnCTG) and the RNA binding protein, Igf2bp1. These data preliminarily implicate the differentiation-induced tsGlnCTG in promoting *c-Myc* degradation by sequestering Igf2bp1, a protein required to prevent *c-Myc* endonucleolytic cleavage [51]. Notably, novel putative interactions between tRFs and several proteins were also reported in this study [50]. This may suggest that multiple layers of tRF regulation could be necessary to determine the phenotypic changes observed in embryogenesis. Building on evidence that RNA modifications affect tRF interactomes [34], more efforts will be necessary to delineate how the presence of individual chemical marks impacts the assembly and function of tRF-protein complexes and comprehensively assess the biological implications of these interactions for pluripotency and fate commitment.

## tRFs as novel regulators of haematopoietic stem cell fate and immune response

**Figure 3.**
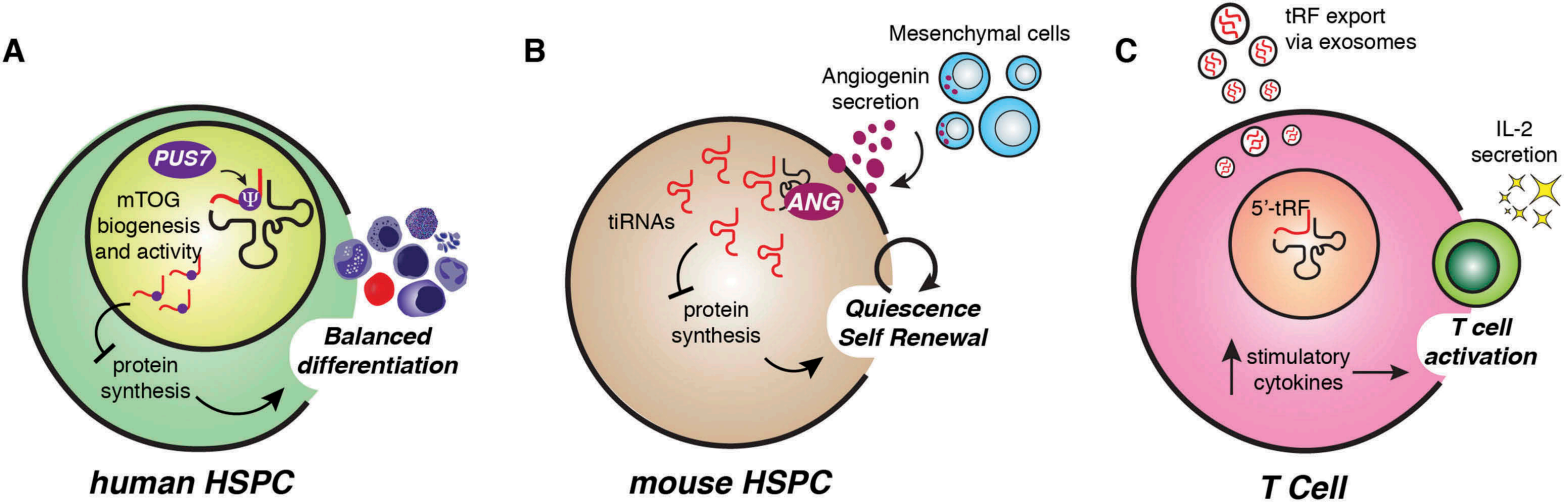
tRFs regulate self-renewal, differentiation and activation of haematopoietic cells. (A) Pseudouridine synthase 7 (PUS7) regulates biogenesis and activity of specific 5ʹ-tRFs, namely mTOGs. In human HSPCs, mTOGs ensure accurate protein synthesis levels and haematopoietic differentiation. Loss of PUS7 and mTOGs leads to aberrant stem cell growth and impaired multi-lineage commitment. (B) Angiogenin is released by MSCs in the bone marrow niche and accumulates in HSPCs to promote tiRNAs biogenesis, maintain low protein synthesis level and stem cell quiescence. (C) Activated T lymphocytes secrete inhibitory 5ʹ-tRF pools to enable production of co-stimulatory cytokines such as IL-2 and immune activation.

There is a growing appreciation that tRFs may share important regulatory functions in adult haematopoietic stem and progenitor cells (HSPCs) [34,52,53], multipotent cells endowed with self-renewal potential that produce all blood lineages throughout life [54,55]. Remarkably, high levels of the pseudouridine ‘writer’ PUS7 in human HSPCs were shown to control protein synthesis and haematopoietic differentiation through activation of mTOGs [34]. Specifically, PUS7 depletion resulted in low mTOG levels that were accompanied by abnormal increases of global translation rates in HSPCs. These molecular dysfunctions led to impaired haematopoietic differentiation *in vitro* and *in vivo* (Fig. 3A). Significantly, we found that PUS7 was frequently altered in myelodysplastic syndrome (MDS), a collection of clonal haematological disorders associated with HSPC dysfunction and high risk of leukaemia transformation [56,57]. Based on previous studies illustrating that HSPCs are highly sensitive to perturbations of protein synthesis [58–60], additional work using *in vivo* models and patient-derived primary cells will be necessary to explore the potentially broad clinical implications of impairments in PUS7 and mTOG in leukaemogenesis. The contribution of tRNA modifications for mammalian haematopoiesis is further supported by findings that *Dnmt2* knockout (*Dnmt2^-/-^*) mice were characterized by HSPC defects [53]. Complete loss of Dnmt2-dependent m^5^C activity on its substrates tRNA-Asp, tRNA-Val and tRNA-Gly was concomitant to a significant increase of tRF levels in the bone marrow (BM) of these mice, which was in line with previous research performed on Dnmt2 mutant *Drosophila* [22]. Interestingly, Dnmt2-depletion in mice did not globally perturb protein synthesis rates but rather affected specific mRNAs through reduced translation fidelity caused by loss of tRNA-Asp methylation [53]. While this suggested that most of the phenotypic effects in *Dnmt2^-/-^* mice were likely the result of translational changes, the potential impact of elevated tRF levels due to Dnmt2 loss in haematopoiesis remains completely unexplored. Thus, additional work will be necessary to unambiguously differentiate the contribution of altered tRF abundance in stem cells from the complex phenotypes observed upon depletion of Dnmt2 and other tRNA modifying enzymes.

A potential role for tRFs as haematopoietic niche modulators was revealed by evidence that angiogenin differentially affected specific populations of stem and progenitor cells [52]. Initial observations indicated that angiogenin expression was different between pools of mesenchymal stromal cells (MSCs) in close proximity to HSPCs, suggesting specialized functions of this secreted RNase in haematopoietic cells [61]. This was subsequently explored using angiogenin knockout (*Ang^-/-^*) mice that displayed abnormal expansion of long-term haematopoietic stem cells (LT-HSCs) resulting from the angiogenin-deficient BM microenvironment. At the molecular level, angiogenin-depleted HSPCs showed significant higher global protein synthesis rates, which were modulated upon transduction of angiogenin or addition of downstream-regulated tRFs including tiRNA-Gly-GCC halves [52] (Fig. 3B). This further highlighted that angiogenin pro-regenerative effects might rely on the repressive effect of specific tiRNA on HSPC translation. This notion was in part supported by previous studies illustrating that phosphorylated 5ʹ-tiRNAs were shown to induce stress granule formation and suppress global protein synthesis in different cell types [4,20,39]. Nonetheless, additional experiments will be needed to comprehensively address the extent to which sequence specificity, 5ʹ-phosphate and RNA modifications contribute to tRF-mediated translation control in the HSPC compartment. Furthermore, *Ang^-/-^* mice were also characterized by defective proliferation of myeloid-restricted progenitor (MyePro) cells [52]. The authors showed that angiogenin impacted MyePro cell proliferation affecting ribosomal RNA (rRNA) levels without global tRF changes in these cells. These findings suggest dichotomous angiogenin-dependent mechanisms for differential control of distinct haematopoietic cell populations and highlighted a critical contribution to the stem cell niche. Interestingly, angiogenin was shown to enhance the survival and proliferation of different cell types including angiogenic, neuronal and cancer cells [62–65]. Nonetheless, how angiogenin substrate specificity is regulated within different cell types and the direct contribution of downstream tRF-dependent and tRF-independent effects on protein synthesis in stem and somatic cells remains to be fully elucidated.

Additional evidence for tRF-mediated regulation in haematopoietic cells is provided by recent work illustrating that tRFs may repress immune activation in mice [66]. Specifically, it was shown that antibody-stimulated T lymphocytes secreted extracellular vesicles (EV) significantly enriched for 5ʹ-tRFs that were different from those released in resting condition. Moreover, it was proposed that the accumulation of 5ʹ-tRFs was detrimental for T cell activation and this was possibly caused by reduced synthesis of co-stimulatory cytokines such as interleukin 2 (IL-2). The authors concluded that activated T cells preferentially eliminated inhibitory intracellular tRF cargos through EV-mediated secretion to maintain cell fitness and activity [66] (Fig. 3C). It remains unclear whether secreted tRFs are further transferred between haematopoietic cells to exchange information and modulate the immune response. Nonetheless, these studies set the basis for future work investigating the contribution of tRFs for physiological and pathological cell-to-cell communications within the haematopoietic system.

## tRF-mediated protein synthesis control in skin and neuronal stem cells

**Figure 4.**
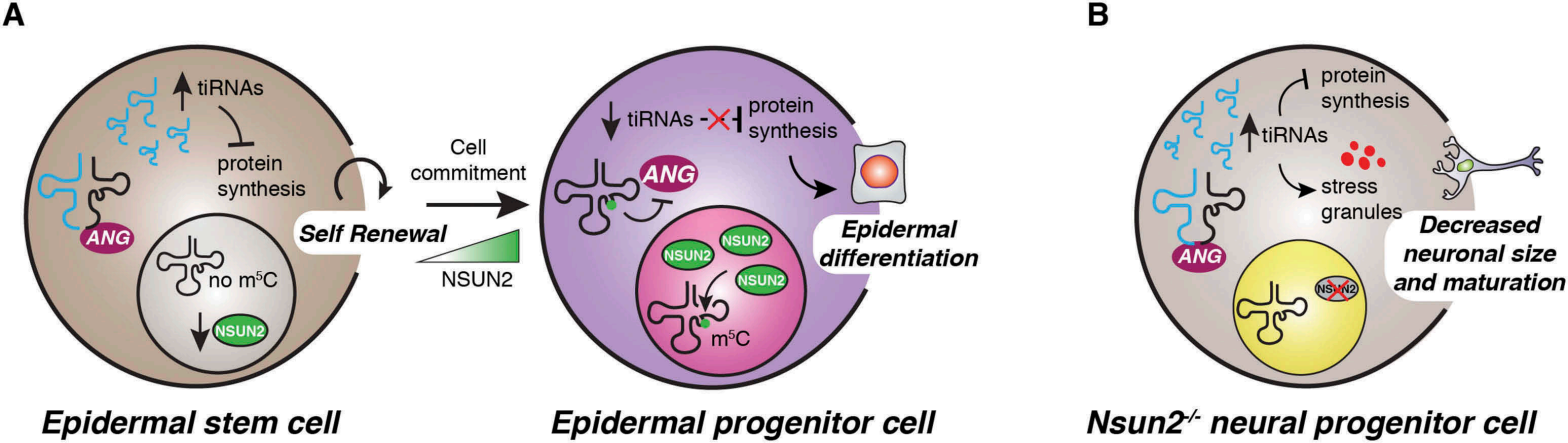
Dynamic tRNA methylation impacts tRF biogenesis and directs stem cell fate. (A) Nsun2 and Dnmt2-mediated m^5^C protects tRNAs from angiogenin cleavage. Low levels of NSUN2 in mouse and human epidermal stem cells lead to accumulation of tiRNAs that repress protein synthesis. Conversely, epidermal progenitors up-regulate Nsun2 to inhibit angiogenic-mediated tRNA cleavage and promote epidermal differentiation. (B) *Nsun2*-depletion leads to neurological disorders caused by accumulation of stress-induced tiRNAs *in vivo. Nsun2*-deficient neuronal cells display increased stress granules assembly, reduced size and impaired maturation. These defects are specifically rescued by angiogenin inhibition that reduces tiRNA levels in these cells.

Somatic stem cells are particularly sensitive to perturbations of protein synthesis and thrive on low translational rates to maintain their undifferentiated state [58,60]. Recent studies showed that specific tRNA modifying enzymes might dynamically affect tRF production across different stem cell compartments and during development [34,41]. In addition to the roles of PUS7 and DNMT2 in accurate embryonic and haematopoietic commitment previously discussed (see above), the function of NSUN2 cytosine-C5 tRNA methyltransferase on modulating tRNA fragmentation during skin development was recently highlighted [41,67]. Specifically, it was shown that Nsun2-mediated m^5^C was critically required to balance stem cell self-renewal and differentiation within the epidermis [67]. *In vivo* studies revealed that Nsun2 expression was highly dynamic during embryogenesis and restricted to subpopulations of committed epidermal stem cells in the adult skin hair bulge [41,67]. Accordingly, skin-specific deletion of Nsun2 led to severe differentiation defects associated with increased stem cell quiescence [67]. Further research illustrated that Nsun2-deficient mouse brains were characterized by tRNA hypomethylation at position C34, C48 and C49 that favoured angiogenin-dependent endonucleolytic cleavage of selective tRNAs and subsequent accumulation of translational inhibitory and stress-inducing 5ʹ-tiRNAs [21]. Increased levels of translational inhibitory 5ʹ-tiRNAs were also observed during skin development. This effect was uncoupled from cell proliferation and was required to maintain skin stem cell homeostasis and function (Fig. 4A). These results further suggested a role for RNA modifications in tRNA fragmentation, which may drive the accumulation of tRFs in stem cells across tissues and organisms even in the absence of external stimuli [34,41,68]. This was also highlighted by studies using a skin tumour model indicating that *Nsun2* deletion repressed protein synthesis to increase cancer-initiating cell self-renewal and promote tumorigenesis [41]. Additionally, rapid downregulation of Nsun2 along with dynamic and site-specific loss of m^5^C on distinct tRNA isoacceptors was observed upon oxidative stress [69]. These stress-induced changes selectively reshaped the tRF landscape to sustain an anabolic cellular state [69]. Future work will be required to investigate whether NSUN2 and tRFs rewire the stem cell metabolome during fate commitment and, when altered, malignant transformation.

NSUN2 is also critically required for accurate development and differentiation of murine and human neural stem cells [21,70]. For example, *Nsun2*-deficient mice were characterized by several neurodevelopmental defects including reduced neuronal cell size and decreased neuron maturation and synaptogenesis [21] (Fig. 4B). These phenotypes correlated with increased cellular stress, accumulation of 5ʹ-tRFs and a consequent reduction of global protein synthesis. Importantly, inhibition of angiogenin could rescue these defects, illustrating the specific effects of Nsun2 on tRF biogenesis and function in neuronal cells [21]. In humans, NSUN2 is highly expressed in early neuroepithelial stem (NES) cells, progenitors endowed with multipotent differentiation capacity towards the neural and glial lineages. Consistent with a central role in development, depletion of NSUN2 in NES cells caused delayed differentiation, possibly through a mechanism involving increased production of tRFs [70]. Accordingly, loss-of-function *NSUN2* mutations have been reported in patients with microcephaly and mental retardation [71–73]. Additional studies are needed to determine the contribution of impairments in tRF epitranscriptomic modifications towards the aetiology of human neurodevelopmental diseases.

## Concluding remarks

The identification of myriad tRNA-derived small RNA molecules harbouring critical roles in development and tumorigenesis has sparked new interest in tRNA research, expanding the complexity of these adaptor molecules beyond their canonical function in translation. Although tRNA fragmentation was identified in the 1970s, tRF biology only recently enjoyed a mechanistic renaissance, which raises important outstanding questions with respect to the biogenesis and molecular pathways controlled by this growing class of small noncoding RNAs (see Outstanding Questions).

Several lines of evidence indicate that different types of mammalian stem cells may be regulated by tRFs under stress and physiological conditions [68]; however, the differential effects of tRFs in stem cell compartments compared to other types of somatic cells need to be fully elucidated. Additionally, the regulatory mechanisms involved in tRF selective processing are still poorly understood. For example, stress-induced tRNA cleavage by the vertebrate-specific RNase angiogenin produces several tiRNAs involved in stemness and differentiation [21,41,52]. Yet, angiogenin is a secreted protein characterized by low sequence specificity [74], suggesting that additional regulatory signals may be necessary to direct cleavage activity within specific stem cell populations. Even less is known about the processes that control the biogenesis of shorter tRF forms across different types of cells. It has been proposed that Dicer-dependent and -independent cleavage events may contribute to 5ʹ- and 3ʹ-tRF production [9,26]. This has raised an important question: how do Dicer and other components of the microprocessor complex survey tRNA integrity? A future challenge will be to delineate the functional overlap with the miRNA and other RNA metabolic pathways to inform the basic mechanisms of tRF biogenesis.

The notion that RNA modifications may be directly involved in tRF biogenesis and function is consistent with tRNAs being heavily post-transcriptionally modified RNA molecules [24]. Several studies have shown that angiogenin-driven tRNA processing is sensitive to m^5^C base modifications installed by DMNT2 and NSUN2 in various cell types including primary epidermal, neuronal and haematopoietic stem and progenitor cells *in vivo* [68]. Furthermore, it has been recently shown that queuosine modification within the wobble anticodon position of specific tRNAs significantly reduces cleavage of tRNA cognates by angiogenin, suggesting that other types of RNA modifications may regulate tRF processing in mammalian cells [23]. Findings in embryonic and haematopoietic stem cells illustrate that ψ is important for tRF biogenesis and function through a mechanism that involves fine-tuned modulation of translation initiation [34]. Therefore, it is tempting to speculate that ψ, m^5^C and possibly other types of RNA modifications may introduce important secondary structural changes necessary for specific RNA–protein interactions that impact tRF maturation and function as recently proposed for other types of small RNAs [75,76]. Recent progress in single-molecule-sequencing along with remarkable advances in mass-spectrometry approaches to detect nucleotide chemical modifications may offer new opportunities to comprehensively assess the tRF epitranscriptomic landscape and determine the specific effects on development and disease [77–79].

Accumulating evidence indicates that dysregulation of RNA modifications may impact human diseases [80–82]. Recent studies compellingly show that cancer cells may co-opt tRFs to affect gene expression and promote tumorigenesis [30,83]. Furthermore, it has been shown that loss-of-function mutations in tRNA modifying enzymes are associated with neurological defects such as microcephaly and intellectual disability in humans [73,84,85]. Accordingly, loss of tRNA modifiers, including PUS7 and NSUN2, leads to perturbed tRF levels associated with aberrant protein synthesis rates in malignant stem cell populations of aggressive skin and haematological cancers [34,41]. These findings highlight both the explosive growth as well as its limits in understanding these new roles for tRFs, as the mechanistic basis by which tRFs govern protein synthesis in stem and cancer cells are still not fully understood. Balanced protein synthesis is central to cellular processes involved in development and tumorigenesis [86]. Future efforts using genome-wide sequencing methods such as ribosome profiling [87] combined with quantitative mass spectrometry [88] will be necessary to decipher the translation-based programs that are driven by tRFs as opposed to those caused by dysfunctional parental isoacceptor tRNA pools in normal and malignant cells [89,90].

Two interesting studies indicate that tRFs may also be transferred between different cell types during development and in adult tissues [44,45]. As such, it is possible that tRFs may thusly exchange information between different cell populations within the stem cell niche and the cancer microenvironment. Additional intriguing developments illustrate that other species of ncRNAs are fragmented into functional small ncRNAs that can also be secreted into extracellular vesicles and may have a role in cancer progression and stem cell differentiation [91]. Interestingly, tRF levels are associated with disease progression in some cancer types [92]; however, the therapeutic potential of these small RNAs remains mostly unexplored. Their clinical promise may be borne out by recent progress with miRNA-based therapeutics [93,94]. Ultimately, as research is unveiling unanticipated facets of gene expression regulated by tRFs in stem cells, this will provide future opportunities to investigate the contribution of this growing class of small noncoding RNAs in regenerative medicine and cancer biology.

## Outstanding questions

1. What is the tRFome that regulates stemness and cell fate differentiation? More broadly, among more than ~20,000 tRFs identified thus far [95], how many are functional and contribute to stem cell regulation?
2. How is tRF biogenesis controlled? What drives the specificity of angiogenin and other ribonuclease complexes towards distinct isoacceptor tRNAs in different types of stem cells?
3. Which upstream signalling pathways govern tRF activity?
4. How do RNA epitranscriptomic modifications impact tRF-protein and tRF-RNA interactions necessary for modulating stem cell function? Are tRF modifications dynamically regulated?
5. Do tRFs contribute to cell-to-cell communication within the stem cell niche?
6. Can tRFs be employed as novel RNA therapeutic tools or predictive, prognostic biomarkers in disease?
